# Transcriptome Analysis Reveals the Function of a G-Protein α Subunit Gene in the Growth and Development of *Pleurotus eryngii*

**DOI:** 10.3390/jof9010069

**Published:** 2023-01-03

**Authors:** Jixuan Cao, Meijing Sun, Mingming Yu, Yanfei Xu, Jiacheng Xie, Huangru Zhang, Jiayi Chen, Tao Xu, Xin Qian, Shujing Sun

**Affiliations:** 1 College of Life Sciences, Fujian Agriculture and Forestry University, Fuzhou 350002, China; 2 Gutian Edible Fungi Research Institute, Fujian Agriculture and Forestry University, Ningde 352200, China

**Keywords:** *Pleurotus eryngii*, G-protein α subunit, cAMP, transcriptome, growth, development

## Abstract

*Pleurotus eryngii* is a commercially important edible fungus with high nutritional and economic value. However, few functional studies have examined key genes affecting the growth and development of *P. eryngii*. In this study, transformed strains, including over-expression (*PeGNAI*-OE) and RNA interference (*PeGNAI*-RNAi) lines, were constructed to elucidate the role of *GNAI* in *P. eryngii* growth. *GNAI* expression was found to affect the mycelial growth and the number of clamp connections. Moreover, the transformed strains were shown to have higher endogenous cAMP levels, thus affecting amylase and laccase activity. Fruiting experiments showed that *GNAI* expression revealed the formation of *P. eryngii* primordia and the number of buttons, while transcription analysis identified *GNAI* gene involvement in the growth and development of *P. eryngii*. Seven downstream genes regulated by *GNAI* were differentially expressed in *PeGNAI-OE* and *PeGNAI*-RNAi compared to wild type (WT). These genes may be related to mycelial growth and enzyme activity. They were involved in the MAPK signaling pathway, inositol phosphate metabolism, ascorbate, aldarate metabolism, and starch and sucrose metabolism. In summary, *GNAI* performs different physiological functions in regulating the growth and development of *P. eryngii*. Importantly, the molecular mechanisms of *GNAI* regulatory function are relatively complex and need further study.

## 1. Introduction

*P. eryngii*, also known as the king trumpet mushroom or king oyster mushroom, is a member of the *Pleurotus* genus [1]. *P. eryngii* is one of the most valuable edible mushroom species in China and other Asian countries because of its thick flesh, delicious taste, and medicinal value [2]. Previous studies have demonstrated that *P. eryngii* exhibited various biological activities, including antioxidant, immunomodulatory, bacteriostatic, and tumor-inhibitory effects [3,4,5]. Mushroom development of *P. eryngii* can be divided into three stages: hyphal knot, primordium, and fruiting body [6]. In the three phases, some cultivation environmental factors, such as temperature, light, CO_2,_ and humidity, could affect the growth and development of fungus [7,8]. Due to the complex genetics and long life cycle of edible fungi, their growth and development are jointly regulated by internal genes and the external environment. The factors of high cost with low efficiency of factory cultivation seriously restrict the development of *P. eryngii* industry. Research on *P. eryngii* to date has mainly focused on the optimization of growth conditions, the selection of media, and the addition of exogenous biomass-promoting substances. There are few basic studies on the cloning and genetic transformation of *P. eryngii* functional genes, and the molecular mechanism regulating growth and development is still unclear.

Several intrinsic and extrinsic factors affect the growth and development of edible mushrooms through different metabolic pathways [8]. Cyclic adenosine monophosphates (cAMP) are widely found in plants, fungi, and bacteria and play a role in secondary message transmission in cells. cAMP responds to various extracellular stimuli and regulates various intracellular signal transduction pathways [9,10]. Heterotrimeric guanine nucleotide-binding proteins (G proteins) play a key role in the cAMP signaling pathway. The G-proteins are composed of α, β, and γ subunits. According to the similarity of function and sequence, Gα proteins can be divided into four families: Gαs, Gαi, GαQ, and Gα12 [11,12,13]. In fungi, when bound to extracellular ligands, the G protein complex consisting of Gα, gβ, and gγ subunits is activated by G-protein-coupled receptors (GPCRs) [14]. In turn, Gα and Gβγ activate downstream effectors, including adenylate cyclase (AC), mitogen-activated protein kinase (MAPK)s, ion channels, phosphodiesterase, and phospholipase [15]. In fungi, biological processes, such as vegetative growth, conidiation and conidial germination, tolerance to various stresses, and response to environmental nutrient status, could be regulated by G proteins and their downstream regulators, which act as molecular switches [16,17,18]. Valle-Maldona et al. demonstrated that the heterotrimeric G-protein β subunit *Gpb1* is critical to the virulence of the fungus *Mucor circinelloides* and can control mycelial growth under hypoxic conditions through the protein kinase A pathway [19]. Gα positively affects conidial germination, cell wall integrity, and thermal tolerance in *Metarhizium robertsii* [20]. In addition, Gα subunit directly interacts with *PsYPK1* in the oomycete pathogen *Phytophthora sojae*, and the absence of *PsYPK1* leads to a significantly reduction in sporangia and oospore production as well as slower mycelia growth [14]. However, little is known about the molecular mechanism of heterotrimeric G-protein signaling in *P. eryngii*.

In a previous transcriptomic study, the cAMP signaling pathway and *GNAI* gene (encoding the G-protein alpha subunit) were linked to *P. eryngii* growth and development [21]. To further explore the function of Gα, *PeGNAI* over-expression (OE) and RNA interference (*RNAi*) strains were generated using *Agrobacterium tumefaciens*-mediated transformation (ATMT). Wild type (WT) in phenotypes and enzymatic activities was compared to those of transformed strains, and the functional role of *GNAI* in the growth and development of *P. eryngii* was further validated using transcriptomic data. This work established a theoretical basis for further research on the molecular regulatory mechanisms underlying *GNAI* effects on *P. eryngii* growth and development and also provided a reference for commercial cultivation to improve the yield of *P. eryngii* substrates.

## 2. Materials and Methods

### 2.1. Strains Materials and Culture Conditions

*P. eryngii* was cultivated by Zhengrong Agricultural Technology Co., Ltd. (Ningde, China) and maintained on potato dextrose agar (PDA). Plasmid propagation of *Escherichia coli* (Trans1-T1) and *Agrobacterium tumefaciens* (EHA105) were incubated in lysogeny broth (LB) containing kanamycin (50 μg/mL) or rifampicin (20 μg/mL); co-cultured *P. eryngii* was inoculated on induction medium (IM). The co-cultured mycelial blocks were transferred to an SM medium containing hygromycin (50 μg/mL) and cefotaxime (150 μg/mL) to screen for positive transformants [18,22]. According to the method of Chen et al., *P. eryngii* was cultured in the culture substrate until the primordium was formed [23].

According to the *PeGNAI* sequence (KAF9491299.1), a primer pair was designed to amplify the *PeGNAI* sequence from cDNA using PCR. The overexpression (OE) and RNA interference (RNAi) vectors of *PeGNAI* were constructed according to the vector pCAMBIA1301-Hygro (Specialty Edible Mushroom Variety Innovation and Metabolic Engineering Team of Fujian Agriculture and Forestry University, Fuzhou, China). Transformed strains were obtained using ATMT according to the reported method [24]. Total RNA was isolated from the mycelia of the transformed strains, and the expression levels of *PeGNAI* were detected by qRT-PCR [25]. After dark culture at 25 ± 1 °C for 7 days, a fungal cake with a diameter of 9 mm at the edge of each colony was inoculated on PDA medium to obtain a sufficient quantity of *P*. *eryngii* for subsequent experiments. WT, *PeGNAI*-OE, and *PeGNAI*-RNAi strains were used for subsequent phenotypic and genetic analyses.

### 2.2. Measurement of Mycelial Growth Rate

As described previously, mycelial blocks (Ø = 9 mm) from WT, *PeGNAI*-OE, and *PeGNAI*-RNAi strains of *P. eryngii* were placed in the culture medium, and the cultures were stored at 25 ± 1 °C [26]. Mycelia growth was measured every 3 d with an electronic vernier caliper, and the growth rate of mycelia was calculated. Each treatment was repeated in triplicate, and each replicate included three plates.

### 2.3. Ultrastructure Observation of the Mycelia

Sections of WT, *PeGNAI*-OE, and *PeGNAI*-RNAi strains were made after culturing on PDA plates for 7 d. The colony edges were cut into small pieces, soaked in 2.5% glutaraldehyde solution, and fixed overnight at 4 °C. Samples were washed repeatedly with phosphate-buffered saline (PBS) buffer solution (pH 6.8), dehydrated in ethanol, transferred to isoamyl acetate, and dried in a vacuum freeze dryer (FD-1A-50, BIOCOOL, China) [27]. A scanning electron microscope (SEM, ZEISS ULTRA 55 SEM Germany) was used to observe the ultrastructure of *P. eryngii* strains.

### 2.4. Enzyme Activity Assay of Mycelia

The mycelial blocks (Ø = 1.0 cm) of *P. eryngii* were cultured for 7 d, dropped into 100 mL of PDA liquid medium, and incubated for 10 d at 25 ± 1 °C and 110 r/min. Mycelia were filtered and centrifuged at 4 °C and 4000 r/min for 10 min. The supernatant was a crude enzyme solution used to determine enzyme activity.

The reduced sugar content was determined using a slightly modified method [28]. The reducing sugar content was determined by the color reaction of 3,5-dinitrosalicylic acid (DNS) with it, and the absorbance value was measured at the wavelength of 540 nm. Laccase activity was determined by monitoring the rate of 2,2′-azino-bis (3-ethylbenzthiazoline-6-sulfonic acid) (ABTS) oxidizing to its cation radical (ABTS+) at 436 nm. The specific procedure was performed by referring to the method of Sun et al. [29]. One unit (U) of enzyme activity was defined as the amount of enzyme required to oxidize 1 μmol of ABTS per minute. An amylase activity assay was conducted using the DNS method [23]. The absorbance was measured at 540 nm (UV spectrophotometer, UV-1801, RAYLEIGH, China). One enzyme activity unit (U) was expressed as a change of 0.1 optical density value per mL of sample reacted with the substrate for 30 min. The intracellular cAMP concentration was determined using the cAMP ELISA Detection Kit (Nanjing, Gen Script Biotech). All steps were determined by a colorimetric assay in strict accordance with the direction of specifications of each assay kit. The above enzyme activity assays were repeated three times for each experiment.

### 2.5. RNA Sequencing and Data Processing

Total RNA was extracted using TRIzol, according to the manufacturer’s instructions (Invitrogen, Carlsbad, CA, USA). The quality of the isolated RNA was detected by a 2100 Bioanalyzer (Agilent Technologies, Santa Clara, CA, USA). The mRNA was purified, and the cDNA library was constructed according to the method of Illumina RNA seq libraries of Shanghai Meiji Biomedical Technology (Shanghai, China). Sequencing was performed on an Illumina Novaseq 6000. The raw sequencing data were filtered using fastp software to obtain high-quality data (clean data) for subsequent analysis [30]. The filtered data were aligned to the reference genome (reference species: *Pleurotus eryngii*; reference genome version: GCA_015484515.1; reference genome source: https://www.ncbi.nlm.nih.gov/genome/45890?genome_assembly_id=1500182 accessed on 20 February 2022), and the data comparison results were counted and evaluated again after quality control, mainly including sequencing coverage situation analysis. Three biological replicates were used for the RNA-seq experiments.

### 2.6. Quantification of Gene Expression Levels and Differential Expression Analysis

The gene expression level in each sample was estimated using RSEM: the clean data were mapped back to the assembled transcriptome, and the read count for each gene was obtained based on the mapping results. The fragment/kilobase transcript/million mapping reads (FPKM) method was used to identify differentially expressed genes (DEGs) with the DESeq2 R package (1.20.0). The false discovery rate (FDR) was used to correct the *p*-value in multiple trials. The FDR ≤ 0.05 and | log2FC | ≥ 1 were set as the screening thresholds for differentially expressed genes (DEGs).

### 2.7. GO and KEGG Enrichment Analysis

Enrichment analysis was performed using the gene Ontology (GO) function and the Kyoto Encyclopedia of Genes and Genomes (KEGG) pathway. Goatools R package was used to analyze the GO enrichment of differentially expressed genes. Go terms with corrected *p*-value < 0.05 were considered to be significantly enriched by DEGs. The Goatools R package was used to enrich differentially expressed genes in the KEGG pathway statistically.

### 2.8. Validation of RNA-Seq by Quantitative Real-Time PCR

Five genes were selected, and qRT-PCR was used to verify the accuracy of the RNA-Seq expression profile. cDNA was synthesized using HiScript RT SuperMix for qPCR kit (R323-01, Vazyme, Nanjing, China). *β-actin* was used as the internal reference gene, and the primers were shown in Table S1. Gene expression levels were calculated using the 2^−ΔΔCT^ method [31]. All analyses were performed with three biological replicates, and each replicate contained three technical repetitions.

### 2.9. Statistical Analysis

To ensure the precision of the results, all experiments were made in triplicate. The compilation and mapping of experimental data were performed using Microsoft Excel 2013 and Sigma Plot 10.0 software. Statistical software SPSS (20.0) were used to analyze the data. One-way analysis of variance (ANOVA) and the least significant difference (LSD) test was used to examine the significant differences among samples.

## 3. Results

### 3.1. Effect of GNAI on Mycelial Morphology of P. eryngii

**Figure 1:**
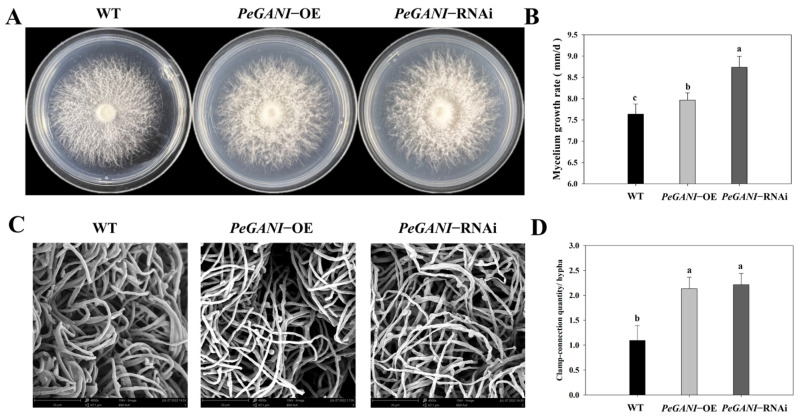
Effects of *GNAI* on mycelial growth in *P. eryngii*. (**A**) mycelial morphology on the PDA plate at 7 d; (**B**) growth rate; (**C**) 4000-fold SEM scans of the microstructure of WT, *PeGNAI*-OE, and *PeGNAI*-RNAi strains; and (**D**) clamp-connection quantity/hypha. Means with different letters are significantly different at *p* < 0.05.

The *GNAI* gene sequence and its open reading frame (ORF) were obtained by PCR amplification. The full length of *GNAI* was 1407 bp, while its ORF was 1065 bp. It contained seven exons and six introns and encoded 354 amino acids (Figure S1A). Phylogenetic tree analysis showed that the Gα protein of *P. eryngii* was most closely related to the Gα protein of *P. ostreatus* (Figure S1D). Overexpression and interference vectors were constructed via homologous recombination, and transformed strains were obtained using ATMT. RT-qPCR results showed that *GNAI* gene expression was upregulated 4.37-fold in the overexpressing OE-2 strain and downregulated 0.65-fold in the interfering transformant RNAi-2 strain compared to the WT (Figure S1E,F). Therefore, these two transformed strains were selected for subsequent experiments. The mycelia morphological structures of WT and the transformed strains of *P. eryngii* are shown in Figure 1. On the plate, the lawn of transformed strains tended to produce flocculent colonies that were thicker in the middle and thinner at the edges (Figure 1A). Compared to the WT strain, the aerial mycelium of *PeGNAI*-RNAi was stronger, and the hyphae were thicker and denser, followed by *PeGNAI*-OE (Table S2). Compared with the WT, the mycelial growth rates of *PeGNAI*-OE and *PeGNAI*-RNAi were 4.46% and 14.42% higher, respectively; this difference was more significant for *PeGNAI*-RNAi (*p* < 0.05) (Figure 1B). SEM observation was performed to confirm the changes of mycelial morphology of *P. eryngii* after *GNAI* mutation. The number of clamp connections for the same number of hyphal branches was: *PeGNAI*-RNAi > *PeGNAI*-OE > WT (Figure 1C).

### 3.2. Effect of GNAI on the Mycelial Enzymatic Activity of P. eryngii

**Figure 2:**
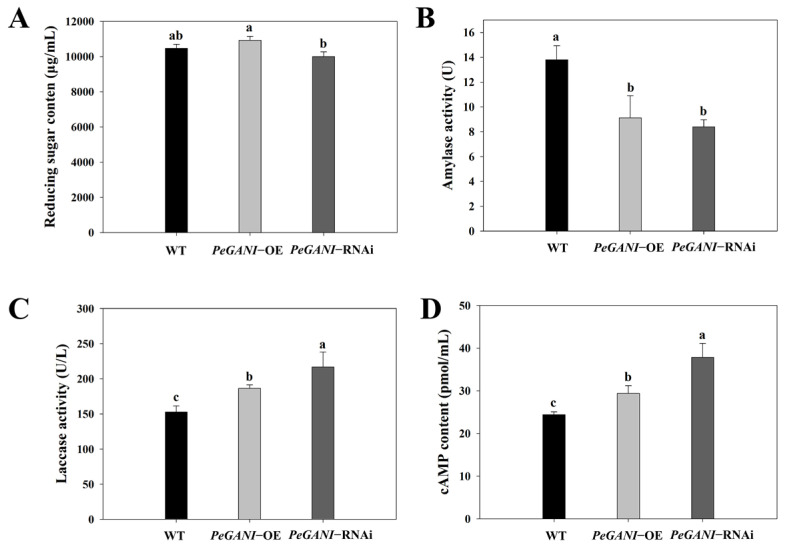
Relative enzyme activity of WT, *PeGNAI*-OE, and *PeGNAI*-RNAi *P. eryngii* strains. (**A**) reducing sugar content (μg/μL); (**B**) amylase activity (U); (**C**) laccase activity (U/L); and (**D**) cAMP content (pmol/mL). Means with different letters are significantly different at *p* < 0.05.

As shown in Figure 2, the reducing sugar content of the *PeGNAI*-OE strains was significantly higher than that of the *PeGNAI*-RNAi strains (*p* < 0.05) (Figure 2A). Compared to the WT, amylase activity was significantly lower (*p* < 0.05) in *PeGNAI*-OE and *PeGNAI*-RNAi, measuring 33.91% and 39.13% less than the WT, respectively (Figure 2B). However, there was no significant difference among transformed strains (*p* > 0.05). At the same time, the laccase activity of *PeGNAI*-OE and *PeGNAI*-RNAi strains significantly increased (*p* < 0.05), and the laccase content of *PeGNAI*-RNAi strains was 1.42 times higher than that of WT strains (Figure 2C). The overexpression or silencing of the *GNAI* gene affected the intracellular cAMP concentrations of *P. eryngii*. As shown in Figure 2D, the intracellular cAMP concentration was significantly higher (*p* < 0.05) in *PeGNAI*-OE (20.40%) and *PeGNAI*-RNAi (55.06%) versus the WT.

### 3.3. Effect of GNAI on Primordium Formation and Button of P. eryngii

**Figure 3:**
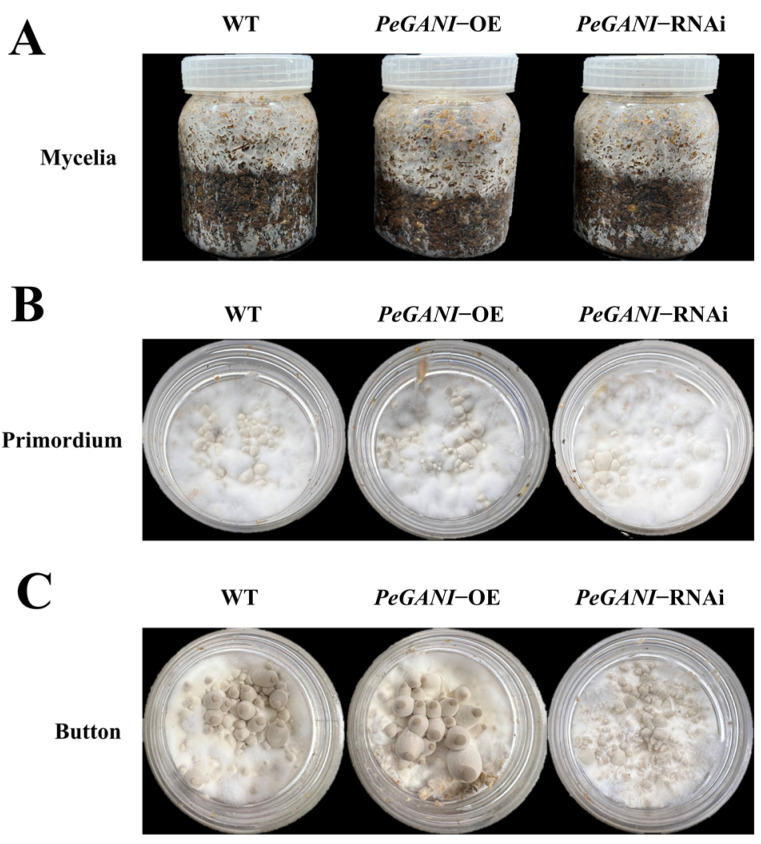
Effect of *GNAI* on primordium formation and button of *P. eryngii* (**A**) mycelial growth in the cultivation bottle for 14 d; (**B**) growth of primordia; and (**C**) growth of buttons.

As shown in Figure 3, the growth rate of *PeGANI*-OE was significantly faster than that of the WT. In contrast, *PeGANI*-RNAi strains showed no significant difference (Figure 3A). Compared to the WT, primordia formation was significantly promoted. The development cycle of the fruiting body was shortened in *PeGANI*-OE strains (Figure 3B). The primordia formation time was slightly promoted in *PeGANI*-RNAi strains but not as significant as the former. The button phase occurred earliest in *PeGANI*-OE, and the small buttons grew well but rarely. *PeGANI*-RNAi strain had the latest button phase, and the small buttons grew thick but weakly. In brief, *PeGNAI* played an active role in developing *P. eryngii*.

### 3.4. Analysis of Differentially Expressed Genes (DEGs)

**Figure 4:**
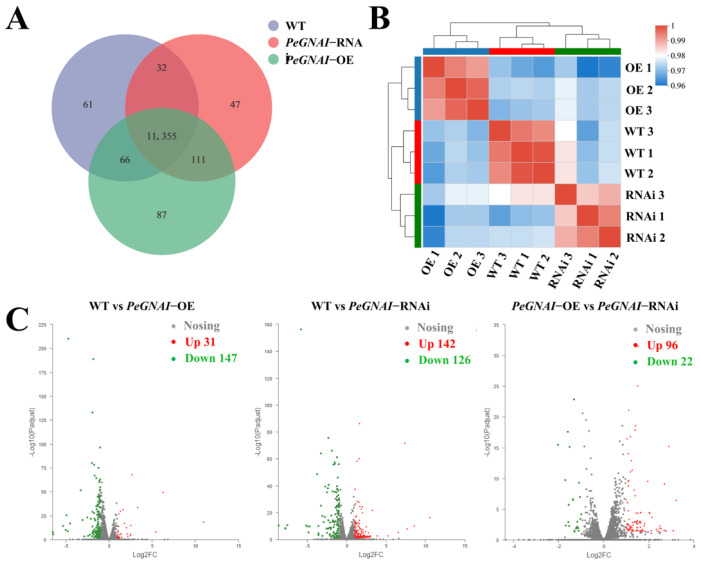
(**A**) Gene expression analysis Venn diagram; (**B**) heat map for inter-sample correlation analysis; (**C**) volcano plot of differentially expressed genes (DEGs) between samples.

DEGs among the groups were identified and annotated to investigate transcript differences of *GNAI* gene during growth and development of *P. eryngii*. As shown in the Venn diagram of the number of DEGs, there were 11514 expressed genes in WT strains, 11619 expression genes in overexpression strains, 11545 expressed genes in interfering strains, and 11355 expressed genes in 3 groups of samples (Figure 4A). To obtain comprehensive gene function information, the entire set of unigenes was annotated via six frequently used databases, including the NCBI non-redundant (Nr) protein database, the Protein family (Pfam) database, the Cluster of Orthologous Groups of proteins (KOG/COG) database, the Swiss-Prot protein database, the KEGG Ortholog (KO) database, and the GO database. All 11456 (90.8%) unigenes exhibited a significant hit with known proteins in the Nr database (Figure S2). The results showed that the correlation coefficient between samples was close to 1, indicating that the correlation between samples was high and the experimental design was reasonable (Figure 4B). Specifically, there were 31 upregulated and 147 downregulated genes in WT strains compared to overexpression strains, 142 upregulated and 126 downregulated genes in WT strains compared with interfering strains, 96 upregulated and 22 downregulated genes in overexpression strains contrasted with interfering strains by P-adjust < 0.05 & |log2FC| ≥ 1 to screen differentially expressed genes (Figure 4C). The above results showed that overexpression of *GNAI* could lead to the down-regulation of most genes. In contrast, the silencing of *GNAI* could lead to the up-regulation of most genes, indicating that *GNAI* act as a negative regulatory gene in *P. eryngii*.

### 3.5. Functional Annotation of Differentially Expressed Genes

**Figure 5:**
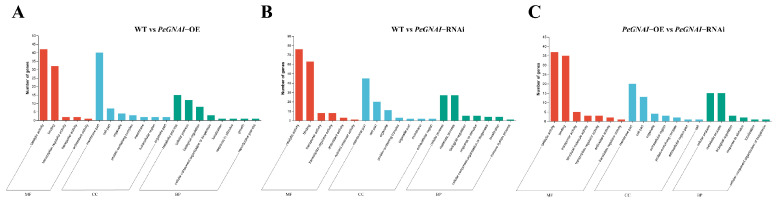
Function annotation column diagram of differential gene GO. (**A**) function annotation column diagram of WT vs. *PeGNAI*-OE group gene GO; (**B**) function annotation column diagram of WT vs. *PeGNAI*-RNAi group gene GO; (**C**) function annotation column diagram of *PeGNAI*-OE vs. *PeGNAI*-RNAi group gene GO. BP: biological processes, CC: cellular components, MF: molecular function.

GO functional annotation and KEGG enrichment analysis were performed to determine these DEGs’ functions. The sequencing results showed that the main biological functions of differentially expressed genes were molecular function (MF) and cell component (CC). Biological process (BP) (Figure 5). The sequencing results showed that BP was mainly concentrated in the metabolic process. Cellular process and biological regulation indicated that *GNAI* may regulate *P. eryngii* mycelial growth through cellular metabolism. CC was mainly concentrated in the membrane, cell, and organelle, which indicated that the expression of *GNAI* affected the cell’s membrane structure and thus influenced mycelia’s growth. MF was focused on binding, catalytic activity, transcription regulator activity, and transporter activity, which indicated that *GNAI* could regulate *P. eryngii* growth by catalyzing and activating various enzymes.

### 3.6. KEGG Enrichment Analysis for Differentially Expressed Genes

**Figure 6:**
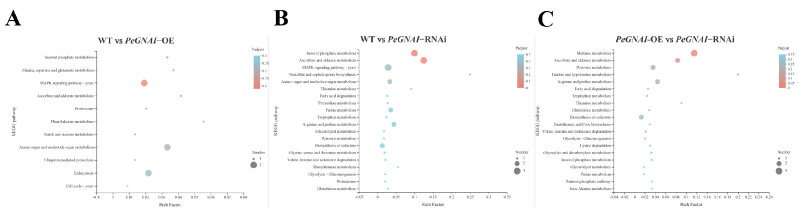
KEGG pathway enrichment analysis for differentially expressed genes in the: (**A**) WT vs. *PeGNAI*-OE; (**B**) WT vs. *PeGNAI*-RNAi; and (**C**), *PeGNAI*-OE vs. *PeGNAI*-RNAi.

To further understand the role of *GNAI* in the mycelial growth of *P. eryngii*, the data of KEGG enrichment, important biological information, the signal transduction pathway and related metabolic pathways were analyzed. Among the DEGs of WT compared to *PeGNAI*-OE, the differentially expressed genes were mainly enriched in MAPK signaling pathway–yeast, an amino sugar, and nucleotide sugar metabolism and endocytosis (Figure 6A). The differentially expressed genes between WT and *PeGNAI*-RNAi were mainly enriched in inositol phosphate metabolism, ascorbate and aldarate metabolism, and MAPK signaling pathway–yeast (Figure 6B). The differentially expressed genes of *PeGNAI*-OE and *PeGNAI*-RNAi were mainly concentrated in methane metabolism, ascorbic acid, aldarate metabolism, pyruvate metabolism, arginine and proline metabolism, and biosynthesis of cofactors (Figure 6C).

### 3.7. Expression of DEGs Related to Mycelial Growth and Development in P. eryngii

**Figure 7:**
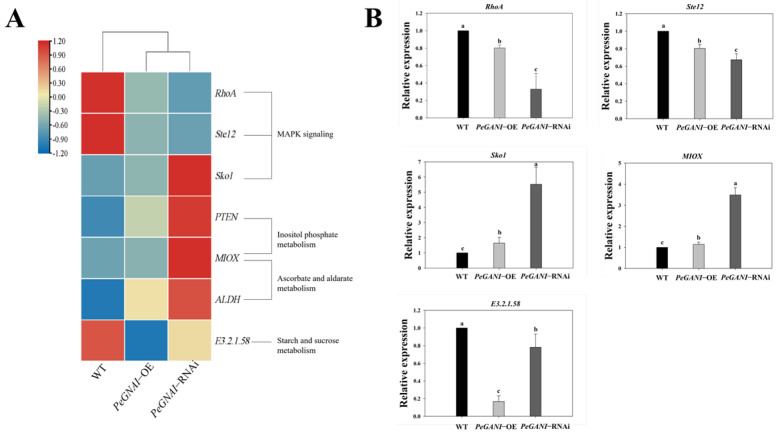
(**A**) Genes involved in mycelial growth and development of *P. eryngii*. The rows in the heat map represent gene names, and the columns represent samples. The color of the heat map cell indicates the gene expression level in different samples; (**B**) validation of DEG by qRT-PCR. Means with different letters are significantly different at *p* < 0.05.

As shown in Figure 7A, the differential genes in related conserved pathways were analyzed. Compared to WT strains, the differentially expressed genes (*RhoA* and *Ste12*) were down-regulated and enriched in the MAPK signaling pathway in *PeGNAI*-OE. Among the differentially expressed genes of WT compared to *PeGNAI*-RNAi, the *Sko1* was enriched in the MAPK signaling pathway, and its expression was upregulated. Analysis of differentially expressed genes in the inositol phosphate metabolism pathway showed that *MIOX* was enriched among the differentially expressed genes of WT compared to *PeGNAI*-OE, and its expression was down-regulated. Among the differentially expressed genes between WT and *PeGNAI*-RNAi, *PTEN* and *MIOX* were enriched in the inositol phosphate metabolism pathway, and both were upregulated. The differential gene analysis in ascorbate and aldarate metabolism showed that *ALDH* and *MIOX* were enriched among the differentially expressed genes of WT compared to *PeGNAI*-RNAi, both of which were upregulated. In the WT comparison of the differentially expressed genes of *PeGNAI*-OE and *PeGNAI*-RNAi, *E3.2.1.58* was enriched in starch and sucrose metabolism pathways and down-regulated. The expression levels of five genes *RhoA*, *Ste12*, *Sko1*, *MIOX* and *E3.2.1.58* were quantified by qRT-PCR to verify our RNA seq results. In conclusion, the present qRT-PCR was consistent with those obtained by RNA-seq (Figure 7B).

## 4. Discussion

King oyster mushroom are commercially grown and widely consumed due to their sensory properties, low calories and high nutritional value [32]. In recent years, with the publication of genomes for several species of edible mushrooms and improvements in transcriptome sequencing technology, edible mushroom research has shifted to the functional studies of signaling pathways and related regulatory genes. Previous studies have shown that Gα subunits regulate the growth and development of fungal species [33]. The silence of two Gα subunit genes in *Ciboria shiraiana*, *CsGPA1* and *CsGPA2*, had no effect on mycelial growth, but they reduced the number of sclerotia and increased the weight of a single sclerotia [34]. In *Aspergillus fumigatus*, deletion of *gpaB* and *ganA* decreased colony growth, while growth was heightened in a *gpaA* deletion strain [35]. In this study, *GNAI* expression was also closely related to the growth and morphological structure of *P. eryngii* mycelia. In *PeGNAI*-RNAi, the mycelial growth rate was significantly higher (than WT; *p* < 0.05), and the mycelial structure was denser with more clamp-connections (Figure 1); this is inconsistent with studies of *Flammulina filiformis*, where the growth rate of *FfGa1*-RNAi transformed strains was significantly reduced. This may be ascribed to the differences in the function of the G-α subunit in regulating asexual growth among different fungal species. Overall, the Gα subunits played an important role in the growth of fungal species.

To further characterize the role of *GNAI* in *P. eryngii*, the key enzyme activities were measured in the mycelial growth phase of transformed strains (Figure 2). Changes in reducing sugar content, amylase activity, and laccase activity could reflect the ability of hyphae to degrade macromolecules and utilize nutrients during growth [28,36]. Hu et al. revealed the significant attenuation of carbon metabolism including the metabolic pathways of starch and sucrose by knocking down the putative Gα-encoding gene *gna1* of coccidia to block the heterologous Gα-cAMP/PKA pathway [37]. *GNAI* silencing resulted in a decrease in amylase activity, which is consistent with the present results (Figure 2B). The silencing of *CsGPA2*, the Gα subunit gene of *C. shiraiana*, resulted in a slight increase in peroxidase and laccase activities. However, the virulence of *CsGPA1* silencing strain was reduced, but peroxidase and laccase activities were normal [34]. In this study, both overexpression and interfering strains significantly enhanced laccase activity compared to WT (Figure 2C). The increase in laccase activity facilitated the utilization of nutrients by *P. eryngii* mycelia, which were consistent with the more rapid growth of the transformed strains in Figure 1. In filamentous fungi, the coupling G-α subunit of the activated G-protein complex regulates cAMP levels in vivo by signaling to adenylate cyclase [38]; cAMP levels play an important role in the cellular response to many extracellular stimuli [39]. Miwa et al. have reported that the mutant lacking G-protein-coupled receptor (*Gpr1*) was involved in the glucose-sensing machinery that regulates morphogenesis and hypha formation in solid media via a cAMP-dependent mechanism [40]. Similar to the present study, Yao et al. found that the transformation of *Pleurotus ostreatus* PC9 with the Gα gene resulted in higher laccase activity and intracellular cAMP concentrations compared to those in WT PC9 [41]. In this study, both *PeGNAI*-OE and *PeGNAI*-RNAi strains caused an increase in endogenous cAMP concentration (Figure 2D). Activation duration of inhibitory G protein (Gαi/o)-coupled receptors affects cAMP accumulation and cAMP-dependent protein kinase activity. That acute activation of Gαi/o-coupled receptors inhibits AC activity, thereby reducing cAMP accumulation. In contrast, sustained activation of Gαi/o-coupled receptors enhances AC activity and increases cAMP when the action of inhibitory receptors is terminated, a phenomenon known as hyperactivation or hypersensitization response [42]. Therefore, it is speculated that AC activity is inhibited and cAMP levels are increased due to reduced expression of *GNAI* in *PeGNAI*-RNAi strains. However, in *PeGNAI*-OE strains, accelerated mycelial growth and increased cAMP concentration may be associated with hyperactivation or hypersensitization of AC. Specifically, the sustained activation of *GNAI* gene would abnormally enhance AC activity, accelerating mycelial growth and increasing cAMP concentration (Figure 1A and Figure 2D). Combined with previous studies, *GNAI* was assumed to affect the activities of amylase and laccase by regulating the level of cAMP, which could affect the mycelial growth of *P. eryngii.* However, some enzyme activities (substrate to product conversion) still require further study.

The growth and development of edible fungi are generally divided into two stages, vegetative growth and reproductive growth, among which the formation of primordium is an important and sensitive stage in the transition from vegetative growth to reproductive growth [23]. In this stage, those mycelia twisted together to form a mycelial community, forming a mass of undifferentiated protoplasm tissue called the primordium or fruiting body [5]. In the mushroom-forming basidiomycete *Schizophyllum commune*, a regulator of G-protein signaling, Thn1, was involved in the development via pheromone signaling that affects physiological interactions [43]. Meanwhile, the present experiment confirmed that G-protein participates in the growth and development of *P. eryngii* by regulating the accumulation of mycelia, the number of primordium formations, and the number of buttons (Figure 3). Consistently, Murry et al. have confirmed the role of G-protein coupled receptors in the development of *Schizophyllum commune* [44].

Transcriptome sequencing analysis for WT, *PeGNAI*-OE, and *PeGNAI*-RNAi strains of *P. eryngii* was performed to investigate the effects of *GNAI* on their downstream signaling pathways and gene expression. In GO with KEGG enrichment analyses, overexpression strains showed down-regulation of most genes (compared to the WT), including *RhoA* and *Ste12* in the MAPK signaling pathway and *MIOX* genes in the inositol phosphate metabolism pathway (Figure 4C and Figure 7A). The silenced strains resulted in the upregulated expression of most genes, including *Sko1* gene in the MAPK signaling pathway, *PTEN* and *MIOX* genes in the Inositol phosphate metabolism pathway, *ALDH* and *MIOX* genes in the Ascorbate aldarate metabolism pathway, etc. (Figure 4C and Figure 7A). MAPK pathways are one of the most important and evolutionarily conserved cellular signaling mechanisms in eukaryotic organisms, including animals, plants, and fungi [45]. The MAPK pathways are involved in transferring the information perceived from extracellular stimuli to cells, resulting in different transcription factors activated in response to them to regulate gene expression. In all species of fungi, the MAPK pathway plays an important role in their physiology and development [46]. Small monomeric GTPases act as molecular switches in all eukaryotic organisms, regulating many cellular processes [47]. Small GTPases are grouped into five subfamilies, with the Rho subfamily most extensively characterized. There are more than 20 members in higher eukaryotes of which *RhoA*, *Rac1,* and *Cdc42* are the most studied [48]. The small GTPase RhoA controls many important cellular processes through its ability to activate multiple downstream effector pathways. They typically cycle between activated GTP-bound and inactive GDP-bound states [49]. Ras GTPases play conserved roles during important developmental stages, including germination, hyphal branching, and asexual development in filamentous fungi [50]. The disruption of the *seb1* gene in strain EMU resulted in morphology defects and more hyphal branching in *seb1* mutant strains. The transcription levels of *rhoA* genes were reduced [51]. The down-regulated expression of *RhoA* was found in both *PeGNAI*-OE and *PeGNAI*-RNAi strains (Figure 7A), which could be responsible for the more luxuriant aerial mycelium of the transformed strains (Figure 1). The homeodomain transcription factor *Ste12* was first found in *Saccharomyces cerevisiae* and was identified as the direct target and function downstream of mitogen activated protein kinase (MAPK) Fus3/Kss1 [52]. Dolan et al. showed that *Ste12* inhibits the growth of filamentous mycelia [53]. The *Ste12* transcription factor has been linked to pathogenicity (via regulation of appressoria) in several fungal species, such as *Metarhizium acridum* and *Setosphaeria turcica* [52,54]. Still, its role in macrofungi has not yet been investigated. Therefore, the down-regulation of *Ste12* might have led to the faster mycelial growth observed here in *PeGNAI*-OE and *PeGNAI*-RNAi (Figure 1). *Sko1* is regulated by the cAMP/protein kinase A (PKA) signaling pathway in response to external stress. Alonso-Monge et al. found that *Sko1* is an important regulator of *Candida albicans* hyphal growth and the oxidative stress response [55]. The enhanced ability of *Sko1* mutants to form mycelia in a liquid medium and the change of colony morphology in different solid media proved the role of *Sko1* in hyphal growth. Silencing *GNAI* increased transcription factor activity and cAMP concentrations in *P. eryngii*, increasing the expression of genes downstream in the MAPK signaling pathway and affecting growth (Figure 2D).

*PTEN* is also known as Tensin-like phosphatase 1 (*TEP1*). Deletion of *FgTEP1* causes *Fusarium graminearum* mycelial growth to become sensitive to lithium, thereby reducing conidial production [56]. TEP1-deficient yeast cells are resistant to lithium and wortmannin, a phosphatidylinositol-3 kinase *(PI3K*) inhibitor, and have a defect in sporulation development [57]. *MIOX* is involved in the glycolytic process and has inositol oxygenase activity, while *ALDH* has ATP-binding and protein kinase activity [58]. In this study, the expression of *PTEN*, *ALDH,* and *MIOX* was significantly increased in the *PeGNAI*-RNAi strain, suggesting that silencing *GNAI* would increase protein phosphatase and inositol oxygenase activities and promote ATP binding to specific proteins, thereby activating PKC, which regulates the gene expression involved in the cAMP signaling pathway and the growth and development of *P. eryngii* (Figure 7A). A differentially expressed gene (*E3.2.1.58*) in the starch and sucrose metabolic pathway is involved in carbohydrate metabolic processes. The *E3.2.1.58* gene was down-regulated in *PeGNAI*-OE and *PeGNAI*-RNAi strains compared to WT strains. This corresponds to the decrease in amylase activity seen in Figure 2B. Similarly, in a pathway enrichment analysis of the pG14 mutant (the putative Gα-encoding gene *gna1*), Hu et al. found 46 down-regulated genes and only 2 upregulated genes involved in the starch and sucrose metabolism pathway [37].

## 5. Conclusions

In this study, the transformed strains of *PeGNAI* were subjected to transcriptome analysis to investigate the functions of *GNAI*. *GNAI* was important in regulating mycelial growth, primordia formation, the number of buttons, cAMP content, enzyme activity, and other aspects. However, the specific effects of the G-protein α subunit on downstream genes needs further investigation, and putative *GNAI* functions should be verified through protein interaction experiments. The results of this study should provide an impetus for further detailed investigations into the role of the G-protein α subunit in hyphal growth, sexual reproduction, and fruiting body development in edible fungi.

## Data Availability

The raw/processed data required to reproduce these findings cannot be shared at this time as the data also forms part of an ongoing study.

## Supplementary Materials

The following supporting information can be downloaded at: https://www.mdpi.com/article/10.3390/jof9010069/s1, Figure S1: A, *PeGNAI* gene structure; B, the secondary structure of Gα subunit gene; C, the tertiary structure of Gα subunit gene; D, the phylogenetic tree of Gα subunit gene; E, RT-qPCR validation of overexpressed transformed strains; F, RT-qPCR validation of silencing (RNAi) transformed strains. Figure S2: Results of gene function annotation. Including the NCBI non-redundant (Nr) protein, the Protein family (Pfam) database, the Cluster of Orthologous Groups of proteins (KOG/COG) database, the Swiss-Prot protein database, the KEGG Ortholog (KO) database, and the GO database. Table S1: Primers used for real-time qRT-PCR analysis. Table S2: Morphological characteristics of *P. eryngii* mycelia.
