# Supplementary material for: Transcriptome Analysis Reveals the Function of a G-Protein α Subunit Gene in the Growth and Development of Pleurotus eryngii
Source: J Fungi (Basel). 2023 Jan 3;9(1):69. doi: 10.3390/jof9010069 (PMC9866537; doi:10.3390/jof9010069)
Supplement: Supplementary file 1 [file jof-09-00069-s001.zip › jof-2116213-supplementary.pdf]

Supplementary Information

Table S1 Primers used for real-time qRT-PCR analysis

Gene name	Primer sequences
β-actin	F: GCTGGTATCCACGAGACAACA
	R: CAAGATAGAACCACCAATCCAAA
RhoA	F: TTGGATAGGTGAAGTGCGACA
	R: TTGCCAGACCGAGTGATTTC
Ste12	F: AACGGTGACGATGGCTACAG
	R: CAGACTTCCTCCGCCTTGA
Sko1	F: GGGTCTCAGCCGCATACA
	R: AGTGGTGTCAGTCCTGTCCCT
MIOX	F: TTTACAAACCACATTGCGGC
	R: ACGAATCATTGCTAAGCCCTC
E3.2.1.58	F: ACGATACCAAAGACCCTTACAAC
	R: TGAAAACAACTCGCCCAAAT

Table S2 Morphological characteristics of *P. eryngii* mycelia

Strain	Growth vigor of aerial mycelia	Edge tidiness of colony	Sturdiness	Density
WT	++	+++	++	++
PeGNAI-OE	+++	++++	++++	+++
PeGNAI-RNAi	++++	++	++++	++++

Note: ++++ indicates a high degree; +++ indicates a high degree; ++ indicates a low degree; + indicates a low degree.

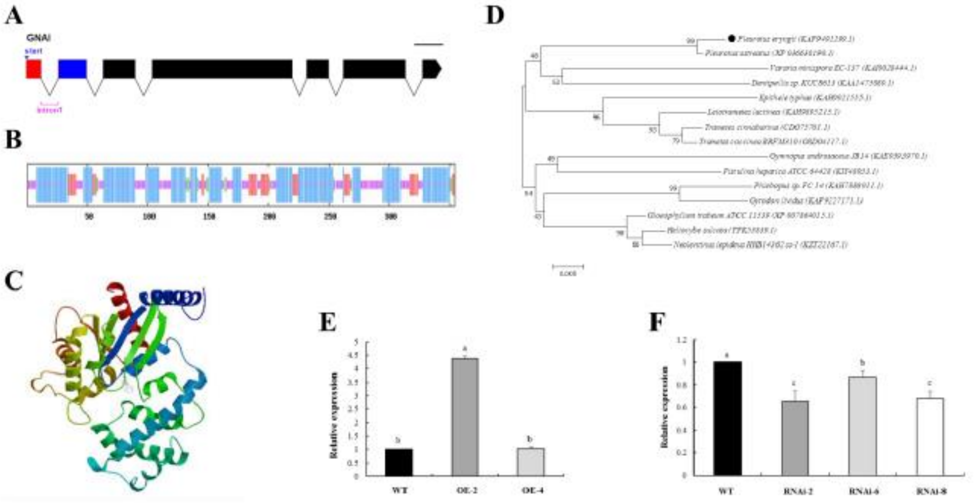

Fig. S1 A, *PeGNAI* gene structure; B, the secondary structure of Gα subunit gene; C, the tertiary structure of Gα subunit gene; D, the phylogenetic tree of Gα subunit gene; E, RT-qPCR validation of overexpressed transformed strains; F, RT-qPCR validation of silencing (RNAi) transformed strains.

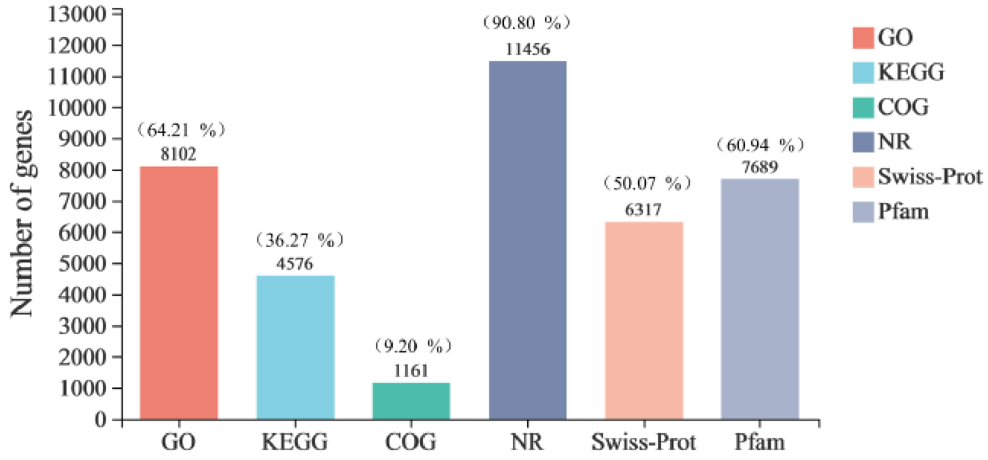

Fig. S2 Results of gene function annotation. Including the NCBI non-redundant (Nr) protein, the Protein family (Pfam) database, the Cluster of Orthologous Groups of proteins (KOG/COG) database, the Swiss-Prot protein database, the KEGG Ortholog (KO) database, and the GO database.
